# Estimating Copy-Number Proportions: The Comeback of Sanger Sequencing

**DOI:** 10.3390/genes12020283

**Published:** 2021-02-17

**Authors:** Eyal Seroussi

**Affiliations:** Institute of Animal Science, Agricultural Research Organization (ARO), HaMaccabim Road, P.O.B 15159, Rishon LeTsiyon 7528809, Israel; seroussi@agri.huji.ac.il

**Keywords:** dye-terminator DNA sequencing, SNP, CNV, CpG, bisulfite sequencing, base editor, RNA editing, replication diode, heteroplasmy

## Abstract

Determination of the relative copy numbers of mixed molecular species in nucleic acid samples is often the objective of biological experiments, including Single-Nucleotide Polymorphism (SNP), indel and gene copy-number characterization, and quantification of CRISPR-Cas9 base editing, cytosine methylation, and RNA editing. Standard dye-terminator chromatograms are a widely accessible, cost-effective information source from which copy-number proportions can be inferred. However, the rate of incorporation of dye terminators is dependent on the dye type, the adjacent sequence string, and the secondary structure of the sequenced strand. These variable rates complicate inferences and have driven scientists to resort to complex and costly quantification methods. Because these complex methods introduce their own biases, researchers are rethinking whether rectifying distortions in sequencing trace files and using direct sequencing for quantification will enable comparable accurate assessment. Indeed, recent developments in software tools (e.g., TIDE, ICE, EditR, BEEP and BEAT) indicate that quantification based on direct Sanger sequencing is gaining in scientific acceptance. This commentary reviews the common obstacles in quantification and the latest insights and developments relevant to estimating copy-number proportions based on direct Sanger sequencing, concluding that bidirectional sequencing and sophisticated base calling are the keys to identifying and avoiding sequence distortions.

## 1. Introduction

Incorporation of chain-terminating dideoxy nucleotide analogues (ddNTPs) during in-vitro DNA polymerization is the basis of the Sanger sequencing method, named after its first developer Frederick Sanger [1]. Aiming at higher throughput, identical nucleotide resolution, and better signal strength, accuracy, and read length, further developments to the Sanger protocol have implemented fluorescent dye-labeled ddNTP terminators and engineered thermostable DNA polymerases [2]. Today, Sanger sequencing is commercially dominated by capillary sequencers using BigDye^®^ terminators and *Thermus aquaticus* (Taq) FS polymerase (AmpliTaq^®^, ThermoFisher Scientific, Waltham, MA, USA [3]). Commercially available as Thermo Sequenase™ [4], this enzyme is an engineered variant of Taq DNA polymerase that contains two mutations, leading to a much more even peak-intensity pattern. A point mutation (F667Y) in the active site results in less discrimination against ddNTPs. The second amino-terminal mutation eliminates the 5′→3′ nuclease activity of the Taq polymerase. A byproduct of increased intrinsic Taq processivity is increased pyrophosphorolysis, which causes some peaks to lose intensity at rates that vary dramatically depending on the adjacent sequences [5]. To eliminate this problem, sequencing Taq polymerase is formulated with divalent metal cations (Mg^2+^; Mn^2+^ [6]) and another thermostable enzyme named inorganic pyrophosphatase [4]. However, unevenness of peak height is also driven by the rate of incorporation of dye terminators, which is dependent on the dye type, as well as the adjacent sequences [3]. Uneven peak heights decrease the accuracy of base calling, and make the estimation of copy-number proportions of mixed molecular species less reliable and detection of polymorphism more difficult. Indeed, a disparity in peak heights at a polymorphic site in heterozygotes has been demonstrated, i.e., at the site of heterozygosity (5′-CT**C**-3′/5′-CT**T**-3′), height of the cytosine (C) peak was 3.5-fold higher than the thymine (T) peak, whereas when using template DNA with even proportions between the two alleles, a 1:1 ratio would have been expected for this double peak [3]. Overscaled C signals and high background noise have indeed been indicated as the reasons why the approach of direct sequencing failed to gain acceptance as a reliable method for quantification of copy-number proportions from sequencing chromatograms [7]. This gave rise to numerous more complex methods, ranging from quantitative polymerase chain reaction (PCR) [8,9] to labor-intensive and time-consuming cloning-based protocols that involve cloning of PCR fragments, construction of recombinant vectors, identification of positive clones, and counting positive subclones following genotyping/DNA sequencing; or, it involves counting spots of next-generation sequencing (NGS) technologies and calculating the copy-number proportions based on depth of coverage [10]. However, each step of a complex method introduces a bias that might complicate quantification, leading researchers to rethink whether rectifying distortions in sequencing trace files and using direct sequencing for quantification would allow comparable accurate assessment of DNA copy-number proportions [7]. Bearing this approach in mind, this commentary is a review of the latest insights and developments relevant to estimating copy-number proportions based on direct Sanger sequencing.

## 2. Single-Nucleotide Polymorphisms (SNPs) and Indels

Sequencing diploid DNA to detect substitution of a single nucleotide at specific positions in the genome may be regarded as a subcase of analyzing copy-number proportions with an expected 1:1 ratio. Automated direct DNA sequencing of PCR products with BigDye-terminator chemistry has been proven to yield superior quality data compared to dRhodamine terminators and is now the most widely used approach [11]. As the pattern of dye-terminator incorporation is dependent on the local sequence context, inaccurate base calling due to uneven peak pattern remains a problem when sequencing with BigDye terminators. In the local sequence context, the two bases immediately 3′ to the substituted nucleotide are the most influential [3]. In most cases, their influence is similar on both alleles, allowing the 1:1 ratio to be maintained in the double peak of the heterozygosity site. However, a few cases, such as 5′-YT**C**-3′ and 5′-NT**T**-3′, result in large and small peaks at the 3′ base, respectively [3]. In such cases, the peak height could potentially differ 10-fold [3], dropping the T peak to background level and rendering it undetectable. Nevertheless, differential dye-terminator incorporation or preferential amplification of one allele during PCR are unlikely explanations for the complete absence of a second peak among heterozygotes, especially in cases where this absence is noted in only one of the two sequencing orientations [12]. Termed replication diode, complete absence has been shown to arise from the presence of stem–loop structures capable of guanine (G)–T wobble-pairing within the tested amplicon. Stabilization of these structures for specific alleles in heterozygous situations mediates the orientation bias by hindering DNA polymerase passage on one strand, while, on the complementary strand, the non-paired adenine (A)–C nucleotide counterparts allow unobstructed replication [13]. Thus, bidirectional sequencing is mandatory for heterozygote detection. When sequencing heterozygous alleles, the different sequence context may also affect the rate at which an allele migrates in the capillary. This means that two heterozygous peaks may not always align and overlap perfectly, with one appearing a head of the other. Moreover, the fluorophores have overlapping spectra that complicate the determination of which one is present. Thus, at positions where two similar fluorophore spectral are present, it may be difficult for the sequencer hardware and software to correctly identify what is present resulting in a systemic error. Nevertheless, inferring relative proportions of DNA variants from Sanger sequencing electropherograms has gained scientific acceptance, and computer software for this purpose has been developed. These include a free desktop application (QSVanalyzer [14], Table 1) that allows high-throughput quantification of the proportions of DNA sequences containing single-nucleotide sequence variants. A notable commercial application for such quantification is the Mutation Quantifier application of the Mutation Surveyor Software package [15]. This application improves the detection of variants with low copy-number proportion (~5%) by comparing the observed and expected peak heights, based on the assumption that intensity ratio of the neighboring same-color peaks is consistent in the samples; this is not always valid, as peak intensities are influenced by their local sequence context [3].

Microindels (1 to 50 nucleotides) are more readily detectable than SNPs, as they result in predictable superimposed trace files following the indel site [16,17]. This detectability has been harnessed to the analysis of heteroplasmic mitochondrial deletions, allowing identification of deleted molecules present in just 5% of the mixture by a specialized tool for detecting low-abundance indels in standard sequence traces [18]. Indeed, the need for techniques to estimate mitochondrial DNA copy number for human clinical diagnosis promoted advances in the detection of mitochondrial DNA heteroplasmic variations (reviewed by [19,20]). A method termed polymorphism-ratio sequencing (PRS) was developed for this purpose based on microfabricated capillary array electrophoresis and the Sanger protocol [21]. However, this method′s limit of minor allele frequency detection was 5%, compared to the limit of similar magnitude (5–7.5%) reported for standard Sanger sequencing or SNaPshot minisequencing with BigDye terminators and capillary sequencers [15,22,23]. For indels, in base strings where the local sequence context is retained, the peak-height ratio between a reference base and its corresponding base in the molecule with the indel could potentially be used to accurately quantify their relative copy-number proportions. However, the need to quantify the efficiency of genome-editing enzymes further promoted the development of more sophisticated methods capable of sensitive analyses of indels in Sanger trace files.

## 3. Base Editing by CRISPR-Cas9 Endonucleases and Nickases

Effective tools based on Cas9 endonucleases and nickases have been developed for the purpose of gene editing with single-base resolution. These base editors rely on the riboprotein complex of Cas9 with a guide RNA to specifically localize these enzymes to the targeted site; their action results in a high frequency of base substitutions and indels [24]. Analysis of the base-editing results typically requires expensive and time-consuming methods, such as Surveyor nuclease assay, subcloning, and NGS [25]. To overcome these limitations, several groups have recently developed computer programs to measure base-editing efficiency from Sanger sequencing trace files, including: Tracking of Indels by DEcomposition (TIDE and TIDER for easy quantification of template-directed CRISPR-Cas9 editing [26]), Edit Deconvolution by Inference of Traces in R (EditR [27]), Inference of CRISPR Edits (ICE [28]), Base-Editing Evaluation Program (BEEP [29]), and Base Editing Analysis Tool (BEAT [30]). These applications, implemented in R and Python, differ in their ability to handle indels and base substitutions (Table 1). The Python versions, developed more recently, add capacity, different statistics, and graphical output. It should be noted that although these tools specifically target analysis of CRISPR-Cas9 outcomes, their use can be easily adapted to the quantification of mixed molecular species derived from other sources, such as bisulfite sequencing.

## 4. C Methylation

DNA methylation of C occurs predominantly on CpG dinucleotides in eukaryotes and seldom on non-CpG bases in metazoans, including human embryonic stem and neuronal cells [33]. It has an important role in the transcriptional regulation of numerous physiological processes; thus, rapid and simple detection of DNA methylation is generating growing interest in both academic and pharmaceutical sectors [33]. Bisulfite treatment of DNA efficiently (>99%) converts unmethylated C into uracil (U), which is amplified as T during PCR amplification. Sanger sequencing of these PCR amplicons yields chromatograms that can be utilized to calculate the methylation status of each individual C by assessing the peak ratio between C and T, representing the methylated and unmethylated C in the original template DNA, respectively [7]. Using the BigDye terminator kit and a series of optimization strategies and techniques, it has been shown that the ratio between C and T peak heights measured by Chromas software can accurately represent copy-number proportions between these bases, solving the problems of methylation quantification by direct bisulfite-PCR sequencing [7]. Yet, as the fluorophore dyes labelling C and T have close emission spectrum maxima (dROX 595 nm and dTAMRA 618 nm, respectively), this analysis might be complicated when their respective peaks are not perfectly aligned. Better peak-height predictability in CpG results can be obtained if sequencing is performed from the reverse orientation, which also yields CG, but always retaining the complementary C preceding the methylation site as the most influential residue in the local sequence context (Table 2). As Chromas provides a "Reverse" button, sequence traces that were performed in one orientation can be presented as if obtained from the other orientation. In such a presentation, it would be expected that in the case of even proportions, the T peak will be larger than the C peak, and indeed up to 30% overscaling of T peaks has been observed for the trace chromatogram of 50% expected values [7]. Thus, interpreting the C:T peak ratios obtained in both sequencing orientations while considering the expected C:T peak-ratio bias reported in the literature may provide a better estimation of C-methylation status. Without correcting for the peak-ratio bias, direct bisulfite sequencing is not considered reliable for quantification [34].

## 5. RNA Editing

There are two known types of messenger RNA editing: adenosine-to-inosine (A-to-I), which is common in all animals; and cytidine-to-uridine (C-to-U), which is rare in mammals but commonly seen in plants. In reverse-transcribed PCR-amplified cDNA, these edits are visualized as A-to-G and C-to-T base substitutions, respectively [35]. Using Sanger chromatograms, determination of the proportion of A-to-I editing is based on measuring the heights of the A and G peaks at the edited position and then dividing the height of the G peak by the sum of the A and G peak heights at this site. Peak heights can be measured automatically using a peak-calling program such as BioEdit or Chromas [36]. An automated tool is currently under development, based on an easy validation method for detecting and quantifying RNA editing from Sanger sequencing (Table 1, [32]). However, users are required to manually trim the 5′ and 3′ ends of the trace file to reduce noisy sequencing. Moreover, with no correction for peak-ratio bias, quantification of RNA editing from direct sequencing will be less reliable in base strings that are prone to this bias.

## 6. Copy-Number Variations (CNVs)

**Figure 1:**
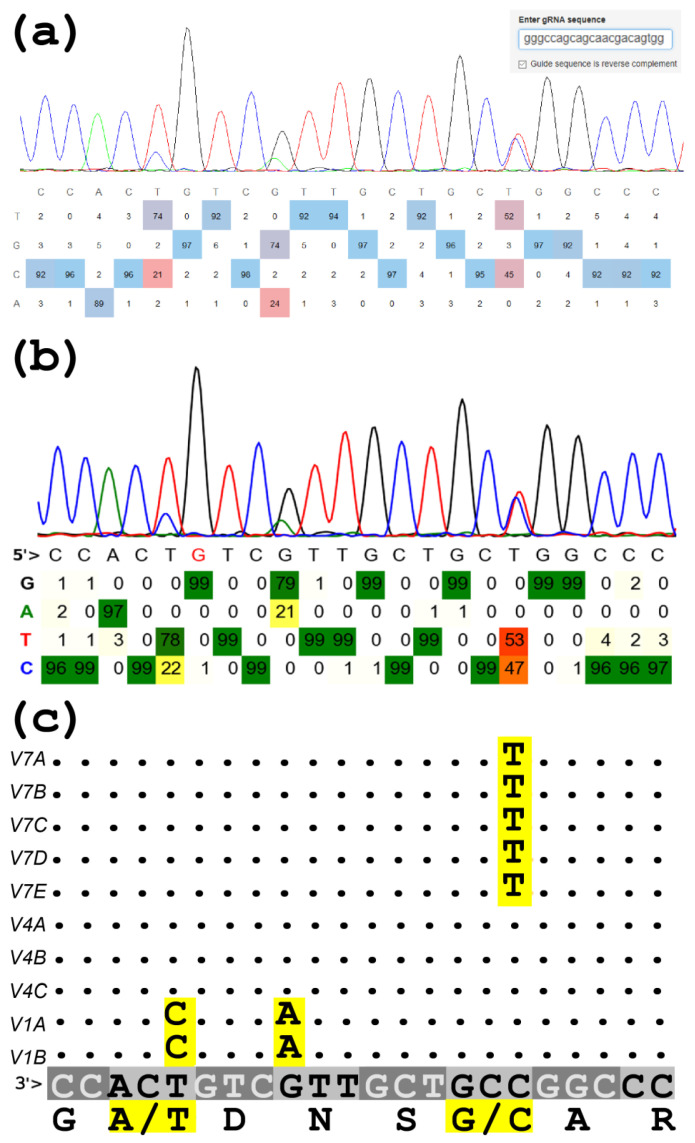
An example of estimating gene copy-number proportions based on sequence chromatograms of the third exon of bovine gene *CD32*. (**a**) Application of EditR, an R program for base-editing quantification [27]. (**b**) Application of BEAT, a Python program for base-editing quantification [30]. (**c**) An allele model based on DNA-sequencing that follows a previously published Figure 4 [40] but in the reverse orientation. Dots indicate similarity to the consensus sequence of 10 allele variants predicted by the assembled sequences and counts of this sire’s DNA-Seq reads. Putative amino acid translation is given below the consensus sequence, in which codons are annotated by alternating font and background color. Nucleotide and amino acid variations are highlighted in yellow. The presented trace file was obtained from sequence analysis using BigDye terminators kit 3.1 (Thermo Fisher Scientific, Waltham, MA, USA) run on an ABI3730 Automated Sequencer; and analyzed by Sequencing Analysis Software 5.3.1 with KB Basecaller v1.4.

CNVs are common variations in chromosomal structure that play an important role in phenotypic variation and genetic disease; SNP genotyping methods that offer independent fluorescence intensities for two alleles can be used to estimate copy-number proportions between copies of segmental duplications [37]. Thus, using Sanger sequencing (AB1) or fragment analysis (FSA) trace files, investigation of peak-height ratio of SNPs within base strings that do not induce peak-ratio bias has been reported as an accurate tool for quantifying gene copy-number proportions [38,39,40]. As demonstrated in Figure 1, peak-calling programs and web tools designed to quantify CRISPR-Cas9 base editing from Sanger sequencing can readily be used to estimate copy-number proportions in CNVs. These tools were tested with an original trace file (Sample4491478.ab1, Supplementary Materials) that had been used to construct the 10 gene copy model of bovine *FCGR2* (*CD32*, Table 3 and Figure 1) [40]. Note that this specific trace file was generated from the reverse orientation and was presented as if performed from the other orientation using the GAP4 assembly program [41]. All tools yielded similar quantifications, with the BEAT tool being slightly closer to the expected values due to the different background-subtraction algorithm used (Table 3). 

Figure 1 also demonstrates the unevenness of the peak heights resulting from the increased rate of ddGTP dye-terminator incorporation in the base string of 5′-CTG-3′, which occurred three times in the presented sequence window. In the left instance of this base string, the G peak is overscaled (170%) compared to right-most G peak (within a 5′-YGG-3′ string). Apparently, such overscaling is not corrected for by the KB basecaller. It has been previously reported that unlike most G peaks, the strength of G peaks in the 5′-CTG-3′ sequence increases disproportionately under certain reaction conditions, such as elevated concentrations of Mn^2+^ [6]. The recently developed basecaller PeakTrace™ (Nucleics Pty Ltd, Woollahra, Australia) is marketed as a paid enhancement of the Chromas freemium, promising better base calling and improved appearance and read length of DNA-sequencing traces [42]. However, this basecaller also does not address the adjustment of peak overscaling in base strings such as 5′-CTG-3′ and thus, estimation of gene copy-number proportions in CNVs using single-base variation might also be biased when using PeakTrace. This was demonstrated by an in-depth analysis of mixed base calling using mussel mitochondrial DNA in which both maternal and paternal genomes are present [43]. The demonstration compared the chromatograms generated by the KB and PeakTrace basecallers in a sequence window that included eight base variations, seven of which indicated a similar proportion between the maternal and paternal types whereas in a single occurrence at the G-to-A variation, the overscaled (~260%) G peak in a 5′-CTG-3′ string unexpectedly suggested otherwise [42]. Thus, neither basecaller corrects for this overscaling artifact. Nevertheless, such peak-height bias is readily detectable by bidirectional sequencing and therefore, analysis of gene-copy proportions based on fluorescence intensities for CNV alleles selected in nonbiased base strings may yield more accurate results compared to other complex methods, because each step of a protocol introduces its own bias, and Sanger direct sequencing has a simple protocol.

## 7. Conclusions

Recent developments in estimating copy-number proportions based on direct Sanger sequencing indicate that such quantification is gaining in scientific acceptance. Best quantification is achieved when the mixed DNA molecular species involve microindels for which superimposed trace files of the indel site allow peak-height ratio analysis. Frequent indels are associated with outcomes of CRISPR-Cas9 base editing and with analyses of mitochondrial heteroplasmy; and the need to quantify these sparked the development of new software tools to infer copy-number proportions from Sanger trace files. As the rate of incorporation of dye terminators is dependent on dye type, the adjacent sequence string and the secondary structure of the sequenced strand, such inference is less acceptable in cases of single-base variations. Frequent observation of overscaled C signals renders direct bisulfite sequencing unreliable for quantification of CpG methylation. However, use of peak-height analysis of a single site has been reported to successfully determine gene copy-number proportions in CNVs and accurately quantify RNA editing. Thus, identifying and rectifying distortions in sequencing trace files may further promote the use of direct Sanger sequencing for quantification. Aside from consulting the available literature presenting characterized base strings associated with distortions, bidirectional sequencing is an easy way to identify and avoid these local sequence effects. Commercial companies have not disclosed the algorithms of their basecallers, which are mostly marketed as part of the software that operates capillary sequencers. However, it is apparent that despite high predictability of distorting effects on peak intensities, current basecallers focus on the identification of bases and not on reporting their relative proportions in mixed DNA templates. This emphasizes the need for base-calling algorithms that can take into account the effects of base terminators on incorporation rates. As different rates are generated by the ever-changing microenvironments of different salt and DNA concentrations, using sophisticated methods, such as neural networks, for base calling [44] may provide the solution for identical nucleotide resolution, which would reflect their true copy-number proportions.

## Figures and Tables

**Table 1:** Free software for estimating copy-number proportions based on Sanger sequencing.

Software	Implemented in	Use Focus	Download/Tool Site	Web Tool	Reference
QSVanalyzer	VB.Net	Single-Nucleotide Polymorphisms (SNPs)	http://dna-leeds.co.uk/qsv/download.php (accessed on 16 February 2021)	N	[14]
BioEdit	C++	SNPs	https://bioedit.software.informer.com/ (accessed on 16 February 2021)	N	[31]
Chromas	C++	SNPs	http://technelysium.com.au/wp/chromas/ (accessed on 16 February 2021)	N	Freemium
TIDE/TIDER	R	CRISPR indels	https://tide.nki.nl/ (accessed on 16 February 2021)	Y	[26]
ICE	Python	CRISPR indels	https://ice.synthego.com/#/ (accessed on 16 February 2021)	Y	[28]
EditR	R	CRISPR SNPs	https://moriaritylab.shinyapps.io/editr_v10/ (accessed on 16 February 2021)	Y	[27]
BEEP	Python	CRISPR indels	https://github.com/mitmedialab/BEEP (accessed on 16 February 2021)	N	[29]
BEAT	Python	CRISPR SNPs	https://hanlab.cc/beat/ (accessed on 16 February 2021)	Y	[30]
MultiEditR	R	RNA SNPs	https://moriaritylab.shinyapps.io/multieditr/ (accessed on 16 February 2021)	Y	[32]

**Table 2:** Peak patterns in Taq-FS sequencing traces with 5′-NCS-3′ base strings following [3].

Base String ^1^	G Peak Height	Base String ^1^	C Peak Height
GC**G**	small	GC**C**	average/large
TC**G**	small	TC**C**	ND
CC**G**	ND	CC**C**	ND
AC**G**	small	AC**C**	average/large

^1^ A string of nucleotides in which the 3′ base is in bold type.

**Table 3:** Estimating copy-number proportions of the bovine gene *CD32* based on Sanger sequencing ^1^.

Variants	Expected	EditR	BEAT	Chromas/BioEdit	QSV
T/C	4	3.52	3.55	3.50	3.54
G/A	4	3.08	3.79	3.08	3.03
C/T	1	0.87	0.89	0.87	0.87

^1^ The ratio between the *V4* type and other types is given based on the variations displayed in Figure 1C.

## Data Availability

Please refer to suggested Data Availability Statements in section “MDPI Research Data Policies” at https://www.mdpi.com/ethics (accessed on 16 February 2021).

## Supplementary Materials

The following are available online at https://www.mdpi.com/2073-4425/12/2/283/s1, Trace file S1: Sample4491478.ab1.
